# Endoplasmic Reticulum Stress Is Associated with the Mesencephalic Dopaminergic Neuron Injury in Stressed Rats

**DOI:** 10.1155/2021/7852710

**Published:** 2021-09-08

**Authors:** Shiba Niu, Weibo Shi, Yingmin Li, Shanyong Yi, Yang Li, Xia Liu, Bin Cong, Guanglong He

**Affiliations:** ^1^College of Forensic Medicine, Hebei Medical University, Hebei Key Laboratory of Forensic Medicine, Collaborative Innovation Center of Forensic Medical Molecular Identification, Hebei Province, Shijiazhuang 050017, China; ^2^School of Forensic Medicine, Xinxiang Medical University, Xinxiang 453003, China; ^3^Institute of Forensic Science, Ministry of Public Security People's Republic of China, No. 17 Nanli Mulidi, Beijing 100038, China

## Abstract

An increasing number of people are in a state of stress due to social and psychological pressures, which may result in mental disorders. Previous studies indicated that mesencephalic dopaminergic neurons are associated with not only reward-related behaviors but also with stress-induced mental disorders. To explore the effect of stress on dopaminergic neuron and potential mechanism, we established stressed rat models of different time durations and observed pathological changes in dopaminergic neurons of the ventral tegmental area (VTA) through HE and thionine staining. Immunohistochemistry coupled with microscopy-based multicolor tissue cytometry (MMTC) was employed to investigate the number changes of dopaminergic neurons. Double immunofluorescence labelling was used to investigate expression changes of endoplasmic reticulum stress (ERS) protein GRP78 and CHOP in dopaminergic neurons. Our results showed that prolonged stress led to pathological alteration in dopaminergic neurons of VTA, such as missing of Nissl bodies and pyknosis in dopaminergic neurons. Immunohistochemistry with MMTC indicated that chronic stress exposure resulted in a significant decrease in dopaminergic neurons. Double immunofluorescence labelling showed that the endoplasmic reticulum stress protein took part in the injury of dopaminergic neurons. Taken together, these results indicated the involvement of ERS in mesencephalic dopaminergic neuron injury induced by stress exposure.

## 1. Introduction

With the rapid development of modern society, more and more people are in a state of stress as a result of increasing social competition and psychological pressures, which has resulted in a number of undesirable mental disorders and remains a major medical and public health issue [1, 2]. Despite taking attention of researchers and many ongoing studies, its mechanism remains unclear.

The ventral tegmental area (VTA) is a major part of the limbic system that plays a pivotal part in reward prediction [3], motivational arousal [4], and responsiveness to conditioned incentive stimuli [5]. The VTA consists of approximately 90% dopaminergic neurons and several *γ*-aminobutyric acid (GABA) ergic neurons. Previous studies have indicated that chronic stress leads to not only elevation of hypothalamo-pituitary-adrenal axis activity [6], a decrease in hippocampal [7] and cortical [8, 9] pyramidal neurons, but also altered activity of dopaminergic neurons in the VTA [10, 11]. Therefore, the critical involvement of dopaminergic neurons of the VTA in the neural circuit modifications is responsible for various adaptive changes and pathological behaviors, which include mental disorders [12, 13].

The endoplasmic reticulum (ER) is a major locale of protein synthesis, glycosylation, folding, and nascent protein transport [14]. Alterations causing instability of ER lead to the accumulation of misfolded and unfolded proteins, which is termed as endoplasmic reticulum stress (ERS) in a pathological process [15]. Recently, mounting evidences indicated that ERS is involved in the pathogenesis of dysfunction due to ischemia and hypoxia as well as in the process of cell death caused by various neurodegenerative diseases [16, 17].

Given a strong association of the mesencephalic dopamine system with stress exposure, we established different durations of stressed rat models and focused on dopaminergic neuron injury in the VTA as well as changes in ERS proteins. The purpose of the present study was to examine whether the ERS was involved in mesencephalic dopaminergic neuron injury of stressed rats.

## 2. Materials and Methods

### 2.1. Animals

Healthy male Sprague Dawley (SD) rats weighing 220 ± 20 g were purchased from Beijing Vital River Laboratory Animal Technology Co. Ltd. Rats were placed in an environment with a constant temperature of 22°C and a light/dark cycle of 12/12 hours. Experimental rats were fed for one week prior to the experiments to adapt the environment. All animal experimental procedures were approved by the Examination Committee of the Animal Experimental Institution of Hebei Medical University. The experiment was divided into 6 groups: control group, 1 day, 3 days, 7 days, 14 days, and 21 days of restraint stress plus ice-water swimming groups (RS+IS), with 10 animals in each group.

### 2.2. Establishment of Stress Models

RS+IS rat models were established according to published methods [18]. On each day, rats were fixed in a restraint device with no food or water for 6 hours (8 : 00-14 : 00). After resting for 10 min, the rats restrained were placed in an ice water tank for swimming for 5 min. The process lasted for 1, 3, 7, 14, or 21 days. In the meantime, the control rats were kept in cages during the same periods with no food or water. During rest periods of each day, all rats were given food and water *ad libitum*.

### 2.3. Rat Tissue Preparation

Sixty minutes after the last of RS+IS, rats were anesthetized via intraperitoneal injection of pentobarbital (150 mg/kg) and their brains were harvested and fixed with 10% formalin for 48 hours. Fixed tissues were subsequently sectioned and stained, following dehydration, clearing, and paraffin embedding. Consecutive 6 *μ*m thick coronal sections were collected to obtain VTA region. For morphological analysis of VTA, coronal slices were cut at -4.80 mm from bregma, according to the protocol provided by Paxinos and Watson atlas [19].

### 2.4. Immunohistochemical Staining

After deparaffinisation and microwave antigen extraction, sections were incubated in 3% H_2_O_2_ chilled methanol for 30 min. Tissues were incubated with a blocking solution (5% bovine serum albumin in 0.1 M PBS) for 1 h at room temperature. Sections were then incubated with mouse TH-specific monoclonal antibody (1 : 200, Abcam, ab219729) at 4°C overnight. The sections were then incubated with a biotinylated secondary antibody (working fluid, ZSGB-Bio), prior to treatment for 30 min with horseradish peroxidase- (HRP-) binding biotin. Finally, 3,3′-diaminobenzidine (DAB) was used as a color developing agent to visualise antibody localisation. Nuclei were counterstained with hematoxylin.

### 2.5. Double Immunofluorescence Staining

Double immunofluorescence staining was performed as described previously [20]. Monoclonal anti-TH antibody (1 : 100, Abcam, ab219729) was used as the first primary antibody, and monoclonal antibodies against GRP78 (1 : 100, Abcam, ab236050) or CHOP (1 : 100, Abcam, ab240220) were used as the second primary antibody. The whole interesting tissues containing first primary antibody and the second primary antibody were incubated at 4°C overnight. Goat Anti-Mouse Alexa Fluor 488-Conjugated IG (1 : 100, Thermo, A11001) and Goat Anti-Rabbit Alexa Fluor 594-Conjugated IG (1 : 150, Thermo, A32740) were employed as secondary antibodies, and they were incubated at 37°C for 30 min.

### 2.6. Counting of Labelled Positive Cells

The accurate identification of the largest VTA was performed based on the stereotaxic atlas [19]. The serial section technique was used to select one from every five sections from each rat for a total of three sections. Microscopy-based multicolor tissue cytometry (MMTC) is employed to quantify DAB-positive cells [18, 21]. Sections (the entire VTA) were scanned using the TissueFAXS quantitative imaging system coupled to a Zeiss® AxioImagerZ2 Microscope (Jena, Germany), and the number of immunohistochemically positive cells was counted by HistoQuest® (TissueGnostics, Vienna, Austria). This quantification technique can accurately count the number of TH-positive cells in the VTA section.

Cell counting was performed to determine the number of TH^+^ cells exhibiting GRP78/CHOP, as described previously [20]. Rats (*n* = 6/group) were selected for morphological observation and data analysis. The numbers of GRP78^+^-TH^+^ and CHOP^+^-TH^+^ cells in the largest VTA area were counted at 100x magnification. The calculation of the average number of positive cells was performed by two independent investigators who were blinded to the study's conditions.

### 2.7. Western Blot Analysis

Four rats from each group were used for western blot. According to a previously described protocol [20], tissue extracts (50 *μ*g of protein/lane) were loaded into an SDS-PAGE gel, separated by electrophoresis, and transferred to polyvinyl difluoride (PVDF) membranes. The membranes were incubated overnight at 4°C with anti-GRP78 (1 : 200), anti-CHOP (1 : 200), and anti-GAPDH antibodies. Then, incubation with horseradish peroxidase-conjugated goat anti-rabbit/mouse IgG was performed. For exposure of the membranes to X-ray film, the enhanced chemiluminescence system was employed.

### 2.8. Statistical Analysis

Data were imported into the SPSS 21.0 statistical software for statistical analysis. The Kolmogorov-Smirnov test showed normal distribution of the data in all groups (*P* > 0.1). All data were expressed as mean ± SEM. The multiple comparison one-way analysis of variance and post hoc LSD *t*-test were used for statistical analysis. *P* < 0.05 was considered statistically significant.

## 3. Results

### 3.1. Stress Damaged Neurons of the VTA

**Figure 1:**
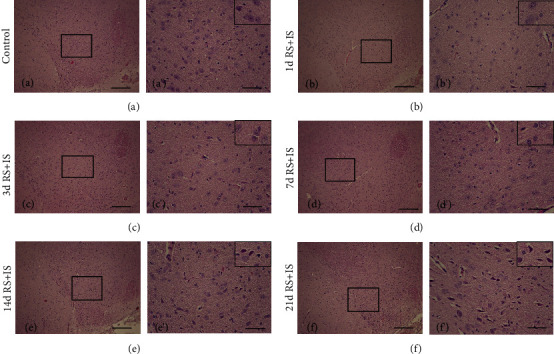
HE staining showed neurons injury in the VTA. a'-f' are magnified of the black frame in (a–f), respectively. Representing pathological changes are shown in the top right corners of a'–f'. The tissue structure and cells were normal in the control group and RS+IS group at 1 day. Edema could be found in the neurons at 3 days and 7 days. However, with increasing stress exposure, some pyknotic neurons were visible at 14 days and 21 days. Bars = 200 *μ*m in (a–f); bars = 50 *μ*m in a'-f'. VTA: ventral tegmental area; RS+IS: restraint stress plus ice-water swimming group.

**Figure 2:**
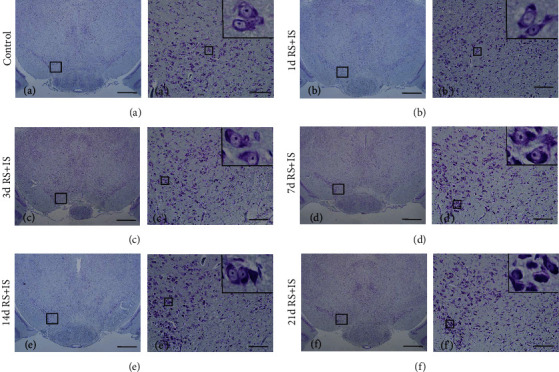
Thionine staining indicated pathological changes of Nissl body and neuron in the VTA. a'–f' are magnified areas of (a–f), respectively. Representing pathological changes in the top right corners of a'–f' are magnified from rectangles. An increase in stress duration led to visible enema, disappearance of some Nissl bodies and pyknosis, and death of some neurons. Bars = 200 *μ*m in (a–f). Bars = 50 *μ*m in a'-f'. VTA: ventral tegmental area; RS+IS: restraint stress plus ice-water swimming group.

HE staining and thionine staining are two traditional methods used to observe pathological changes in neurons. As shown in Figure 1, the tissue structure and cells were normal in the control group, and no significant changes of cells were observed in the RS+IS group at 1 day. Edema could be found in the neurons at 3 days and 7 days. However, at 14 days after stress exposure, scattered pyknotic neurons were detected. In addition, cellular damage was more pronounced at 21 days. The pathological changes of neurons in thionine staining were similar to that in HE staining. As shown in Figure 2, in the control group and RS+IS group at 1 day, there were normal neuronal structures and evenly distributed Nissl bodies in the cytoplasm. With an increase in stress duration, edema was visible and Nissl bodies were unevenly distributed at 3 days and 7 days. At 14 days after stress exposure, Nissl bodies disappeared and several pyknotic neurons were visible. Moreover, cellular damage was more pronounced at 21 days, neurons were pyknotic and dying, and more Nissl bodies disappeared.

### 3.2. Stress Decreased the Number of Dopaminergic Neurons in the VTA

**Figure 3:**
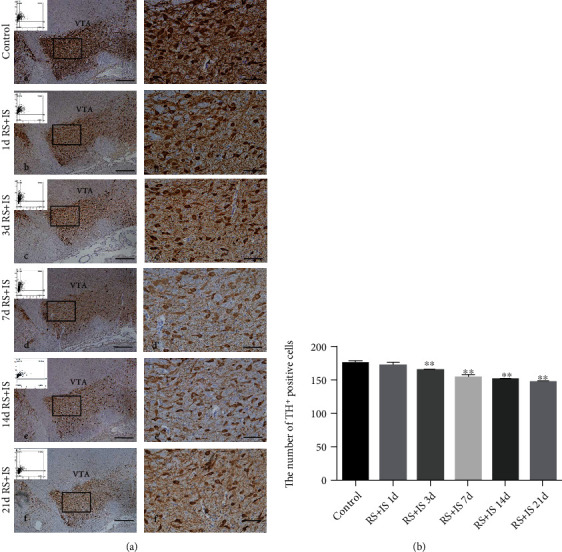
Stress decreased the number of TH-positive cells (dopaminergic neurons) in the VTA. (a) Immunohistochemistry showing TH expression in the VTA. a'–f' are the amplification of the same region of a-f. Representative images of microscopy-based multicolor tissue cytometry (MMTC) are shown in the left corners of a-f. Bars = 200 *μ*m in a-f. Bars = 50 *μ*m in a'-f'. (b) Quantitative analysis of TH-positive cells in the VTA (*n* = 6). Data are shown as mean ± SD. ^∗∗^Significantly different after stress exposure by one-way ANOVA (*P* < 0.01). VTA: ventral tegmental area; RS+IS: restraint stress plus ice-water swimming group.

Tyrosine hydroxylase (TH) is the rate-limiting enzyme in dopamine synthesis [22] that plays a pivotal role in determining dopamine transmitter identity [23] and is usually used to mark dopaminergic neurons [24]. The cytoplasm and synapses of dopaminergic neurons were stained brown, and all regions of the VTA were easily identified (Figure 3). ANOVA for the numbers of TH^+^-positive cells suggested a significant effect after different durations of stress exposure (*F*_(5, 30)_ = 276.971; *P* < 0.001). Post hoc comparisons showed that the number of TH^+^-positive cells was remarkably decreased after stress exposure at 3 days (*P* < 0.05), 7 days (*P* < 0.05), 14 days (*P* < 0.05), and 21 days (*P* < 0.05), while insignificant change was found at 1 day (*P* > 0.05) compared with the control group.

### 3.3. Dynamic Changes of ERS Protein GRP78 and CHOP in the VTA

**Figure 4:**
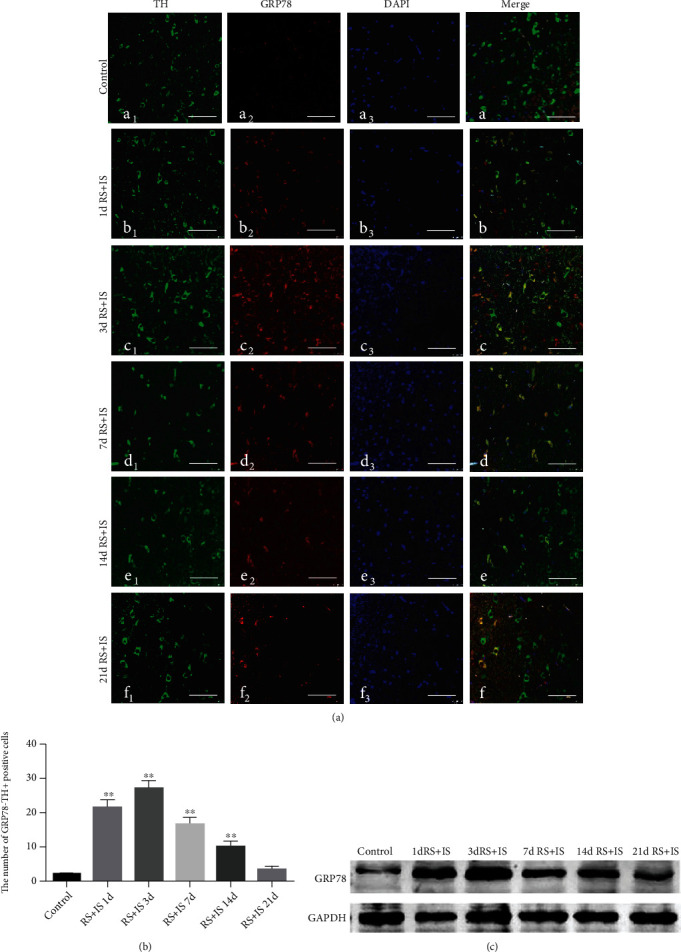
Expression changes of coexisted GRP78 and TH in the VTA. (a) Confocal images showing coronal section n of VTA immunofluorescence labelled for TH (green), GRP78 (red), DAPI (blue), and merged (orange/yellow). Bars = 25 *μ*m. (b) Quantitative analysis of the number of GRP78^+^-TH^+^ cells (*n* = 6). Data are shown as mean ± SD. ^∗∗^Significantly different after stress exposure by one-way ANOVA (*P* < 0.01). (c) Western blot analysis of GRP78 expression in the VTA. VTA: ventral tegmental area; RS+IS: restraint stress plus ice-water swimming group.

**Figure 5:**
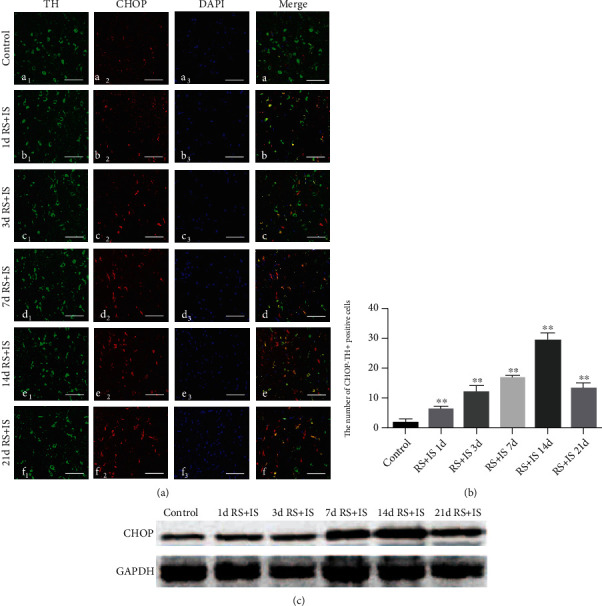
Expression changes of coexisted CHOP and TH in the VTA. (a) Confocal images showing coronal section of VTA immunofluorescence labelled for TH (green), CHOP (red), DAPI (blue), and merged (orange/yellow). Bars = 25 *μ*m. (b) Quantitative analysis of the number of CHOP^+^-TH^+^ cells (*n* = 6). Data are shown as mean ± SD. ^∗∗^Significantly different after stress exposure by one-way ANOVA (*P* < 0.01). (c) Western blot analysis of CHOP expression in the VTA. VTA: ventral tegmental area; RS+IS: restraint stress plus ice-water swimming group.

Double immunofluorescence labelling showed that GRP78 and CHOP proteins were colocalized with dopaminergic neuron marker TH in the VTA (Figures 4(a) and 5(a)). As shown in Figure 4(b), ANOVA for GRP78^+^-TH^+^-positive cells showed significant changes after different time of stress exposure (*F*_(5, 30)_ = 216.947; *P* < 0.001). The number of GRP78^+^-TH^+^-positive cells was markedly increased in the stress group at 1 day (*P* < 0.05), peaked at 3 days (*P* < 0.05), and remained significantly higher at 7 days (*P* < 0.05) and 14 days (*P* < 0.05) compared with the control group; however, no significant alteration was detected at 21 days (*P* > 0.05). The relative level of GRP78 in the VTA analysed by western blot was consistent with the immunohistochemistry results (Figure 4(c)). As depicted in Figure 5(b), ANOVA for CHOP^+^-TH^+^-positive cells indicated significant differences after different durations of stress treatment (*F*_(5, 30)_ = 227.898; *P* < 0.001). Post hoc test revealed that compared with the control group, CHOP^+^-TH^+^-positive cells were significantly increased after stress treatment at 1day (*P* < 0.05), 3 days (*P* < 0.05), 7 days (*P* < 0.05), 14 days (*P* < 0.05), and 21 days (*P* < 0.05). Consistent with the immunohistochemistry results, the relative level of CHOP in the VTA analysed by western blot showed the same trend (Figure 5(c)).

## 4. Discussion

Stress refers to natural responses of the body to different stressors including various environmental, social, and psychological factors. Moderate stress helps body to cope with external risk factors. However, a series of abnormal psychological and physiological changes will occur due to excessive stress. Of late, an increasing number of people are in a state of stress due to social and psychological pressures, leading to mental disorders such as depressed mood, suicidality, and anhedonia [25–27]. In this study, the rat model was successfully established with restraint plus forced ice-water swimming, which could well reflect the effect of complex physical and psychological stress on body.

Mental disorder, a complex condition induced by stress, involves many neuronal circuits. Among the regulation of these neuronal circuits, mesencephalic dopamine system may play a crucial role. The dopamine system plays a critical role in reward prediction [3], motivational arousal [4], and responsiveness to conditioned incentive stimuli [5]. Previous studies indicated that stress could lead to anhedonia in animals [28] that has been associated with dysfunctions in the reward system, particularly in the dopamine system [29]. Stress also resulted in anxiety and depression disorders [30], and neuroimaging, pharmacological, and electrophysiological methods were employed by researchers in humans and animal models, providing support for the presence of dopamine dysfunctions [31]. Particularly, Tye and collaborators [32] indicated that stress-induced abnormal behaviors could be reversed by selective activation of the dopamine system. All these suggested that there is evidence linking the dopamine system with stress. Nevertheless, overwhelming majorities of studies have focused on functioning and metabolic functions, and few pathological studies have concentrated on dopaminergic neuron. Therefore, in the present study, we focused on investigating the pathological changes of mesencephalic dopaminergic neurons under different durations of stress exposure. Our results showed that prolonged stress led to pathological changes in dopaminergic neurons of VTA, such as missing of Nissl bodies and pyknosis in dopaminergic neurons. Moreover, the number of dopaminergic neurons labeled with the specific marker TH was detected, and our findings suggested that there was a significant decrease in dopaminergic neurons in VTA after long-term exposure to stress. These findings indicated that long-term stress exposure could severely damage the dopaminergic neuron and significantly impair its functions, which were in accordance with the previous studies [10, 11]. Any change in morphology of cells could be accompanied by a change in its function. Therefore, these suggested that the dysfunction of the dopaminergic neuron is one of the important causes of mental disorder induced by stress.

The relationship between ERS and injury has attracted the attention of numerous researchers. ERS is practically involved in the mechanism of cell protection; however, excessive ERS contributes to undesirable consequences including cell damage or death. For instance, ERS could activate the caspase family, such as capase-12, and induce cell apoptosis. GRP78 is a specific marker of ERS activation that is merely expressed in the ER. GRP78 determines ERS initiation and plays a vital part in promotion of unfolded protein maturation and maintenance of ER homeostasis [33, 34], suggesting that it plays a cellular protective role. In the present study, GRP78 expression was showing an inverted U type. By 21 days, its expression was not different from that in the control group and significantly lower than other stressed groups. Together, these results indicated that the protective effect of GRP78 on dopaminergic neurons was remarkably undermined due to prolonged stress exposure. In the meantime, a series of downstream signaling pathways that result in undesirable consequences were activated, particularly allowing PERK activation. Activated PERK directly phosphorylates eIF2*α*, inhibiting mRNA transcription and promoting the translocation and activation of ATF4, thus inducing the expression of the transcription factor CHOP [35]. CHOP serving as a key mediator of cell death induced by ERS [36] is poorly expressed under physiological conditions, however notably upregulated when ERS is severe and/or too long. In this study, although we failed to investigate the whole pathway of PERK-ATF4-CHOP, considering that CHOP is a critical approach to ERS leading to cell injury or death [37], we noted a significant increase in CHOP in dopaminergic neurons after stress exposure, indicating in part that ERS was involved in the damage of dopaminergic neurons as well as a decrease in the number of dopaminergic cells. Interestingly, we also found that a small number of CHOP-positive cells were not colocalized with TH. Since VTA is mainly composed of dopaminergic neurons and several GABAergic neurons, ERS may also act on GABAergic neurons indirectly, thereby affecting the activity of dopaminergic neurons. Therefore, further study is needed to explore the specific mechanism. Taken together, these data indicated that the endoplasmic reticulum stress was involved in mesencephalic dopaminergic neuron injury, which might be responsible for occurrence of mental disorders induced by stress.

## Data Availability

The data used to support the finding of this study are included within the article.
